# The legal paradox of uterus transplantation in transgender women: reproductive innovation and regulatory limits after *For Women Scotland*

**DOI:** 10.1093/medlaw/fwag035

**Published:** 2026-10-05

**Authors:** Natasha Hammond-Browning

**Affiliations:** Cardiff Law School, Cardiff University, Cardiff, CF10 3XJ, Wales, United Kingdom

## Abstract

Uterus transplantation (UTx) is a novel reproductive technology that intersects organ transplantation and assisted reproduction. Although births following UTx are a clinical reality, the procedure raises legal questions not anticipated by UK legislative frameworks. This article examines whether UTx may lawfully be provided to transgender women for reproductive purposes under UK law through analysis of the Human Tissue Act 2004, Human Fertilisation and Embryology Acts 1990 and 2008, Gender Recognition Act 2004, and the Supreme Court decision in *For Women Scotland Ltd v The Scottish Ministers*. The article considers whether a transgender woman may lawfully receive a uterus transplant and whether fertility treatment may be provided to facilitate gestation. It argues that the Human Tissue Act 2004 contains no sex-based restriction on organ recipients and therefore does not prohibit transplantation into a transgender woman. However, the reproductive purpose of UTx depends upon access to fertility treatment. Applying the reasoning in *For Women Scotland* generates uncertainty regarding the legislative meaning of ‘woman’. The current statutory framework casts serious doubt upon the legality of providing fertility treatment necessary for gestation. This analysis demonstrates the tension between medical innovation and a legislative framework developed before UTx was contemplated, highlighting the need for legislative clarification.

## I. Introduction

Uterus transplantation (also referred to as UTx) is one of the most recent innovations in organ donation/transplantation and reproductive medicine. As a quality-of-life transplant, UTx stands apart from other quality-of-life transplants as (i) it is life-producing, (ii) it is intended to be a temporary transplant, and (iii) it is performed on essentially healthy individuals. It is now 40 years since the publication of the seminal Warnock Report, which laid the foundation of the UK’s legislative position on human fertilization and embryology, and over 25 years since the publication of the Kennedy and Redfern reports that led to the introduction of the Human Tissue Act 2004.^1^ It is therefore timely to consider the application of these statutes to today’s medical innovations, focussing on UTx.^2^ Furthermore, the recent decision of the UK Supreme Court in *For Women Scotland Ltd v The Scottish Ministers* [2025] UKSC 16 (hereafter *For Women Scotland*) invites attention to transgender access to UTx. Literature points to an interest amongst the medical community in performing this procedure upon a transgender woman, and interest in accessing UTx has also been expressed by some transgender women. The purpose of this article is to analyse and apply the UK’s legislative position on organ donation/transplantation and assisted reproduction, and relevant case law to UTx in transgender women for reproductive purposes, to determine if it is legally permissible to perform in the UK.

This original research goes beyond the existing literature that focuses upon access to organs or access to assisted reproduction. There is limited scholarship bringing together the two legislative areas in conjunction with recent case law and applied to UTx. I argue that it is not illegal to provide the uterus transplant itself to someone who is not a cisgender woman, but that they would currently be unable to access the necessary fertility treatment to fulfil the reproductive purpose of the transplant. This is important knowledge because clinicians need information about the legal ramifications of possible future medical developments, and the transgender community needs to be fully informed about access to UTx, and for expectations to be realistic in light of medical and legal developments. It is acknowledged that a transgender woman receiving a uterus transplant would trigger legal reform^3^; however, the first such transplant would take place under the existing legislative regime, and due to the slow pace of legislative reform, many more could follow. As such, I analyse and apply the existing legislation and case law.

This article analyses two legal issues: first, the ability of transgender women to access and receive a uterus transplant, and second, the legality of providing fertility treatment to assist transgender women to gestate following that transplant. These are determined through an analysis of the relevant legislation and case law, including the recent UK Supreme Court judgment in *For Women Scotland*.

The article is divided into three substantive sections that address the legal issues arising from the potential use of UTx by transgender women. Section II explains briefly the uterus transplant process, who can currently access it, and then demonstrates the interaction and application of the existing regulatory framework governing organ donation, transplantation, and assisted reproduction under the Human Tissue Act 2004 and the Human Fertilisation and Embryology Acts. Section III explores whether transgender women may lawfully access the transplant itself, considering current clinical selection criteria, medical feasibility, equality and human rights protections, and the implications of *For Women Scotland*. Finally, Section IV analyses whether fertility treatment can legally be provided to a transgender woman following transplantation, focusing on the Human Fertilisation and Embryology Acts, the decisions in *McConnell*^4^ and *For Women Scotland*, and the statutory meaning of ‘woman’ within UK fertility law. Through this analysis, the article argues that while a uterus transplant may lawfully be provided to a transgender woman, the current legal framework governing assisted reproduction creates significant barriers to the fertility treatment required to achieve the reproductive purpose of the transplant.

## II. UTx: regulating the process

UTx is a relatively new medical innovation; it first hit the headlines in 2014 with the announcement of the first birth following a uterus transplant.^5^ This procedure crosses medical disciplines and legislative frameworks and raises interesting legal and ethical issues, including but not limited to: selection criteria and access, consent, welfare of children, and resource allocation.^6^

The process of UTx involves two medical disciplines—organ donation/transplantation and assisted reproduction. Within the UK, different legislative frameworks apply to each of these medical disciplines. As Williams and I have noted elsewhere, ‘While UTx itself is an isolated procedure, it occurs as part of a long and arduous process (for all involved parties…)’.^7^ Prior to the transplant, potential recipients will undergo egg retrieval for embryo creation *in vitro* and subsequent storage. The process subsequently involves the donation of a uterus via hysterectomy from either a living or a deceased donor. The uterus is then transplanted into the recipient, who must then take immunosuppressive medication to prevent rejection of the uterus. Provided that the transplant is successful (whereby the uterus is not rejected, and menstruation has started), a single embryo transfer will be performed normally 3–6 months post-transplant. Caesarean section births are performed at least 2 weeks prior to the due date. A second pregnancy may be attempted if desired and medically advised. A hysterectomy is performed either immediately after birth or shortly after.^8^

UTx is currently only offered to cisgender women with Absolute Uterine Factor Infertility (hereafter AUFI) who have ovaries and a sufficient ovarian reserve. Women may have either congenital or acquired AUFI; they have either been born without a uterus, have a non-functioning uterus, or have had a hysterectomy. To date, the majority of uterus transplant recipients have been women with Mayer–Rokitansky–Küster–Hauser syndrome (hereafter MRKH); MRKH is a congenital disorder that affects the female reproductive system. Women with MRKH often have functioning ovaries but have either an underdeveloped or no uterus, with a shortened vagina. MRKH occurs in approximately 1 in 4500 births. Prior to UTx clinical trials commencing, women with AUFI had no options that would have allowed them to gestate, and their only route to have a genetically related child was via surrogacy. Motherhood could also be achieved through adoption or surrogacy utilizing egg donation.

Reasons for pursuing UTx include a desire to experience gestation, to become a mother, moral and/or religious objections to utilizing surrogacy, objections or barriers to accessing adoption, and financial and/or legal barriers to accessing alternative routes to motherhood. Alternative options may also be shrinking. For example, within the UK, non-commercial (non-profit, altruistic) surrogacy is legally permitted; however, very few such non-commercial agencies exist, and all have long waiting lists. In addition, the availability of support from such agencies is decreasing, with one closing in September 2025.^9^

A donation of a uterus from a living donor resulted in the first birth in 2014.^10^ The first birth following a deceased donation was in 2017.^11^ Over 160 uterus transplant procedures have been performed worldwide, with over 80 babies born, and some women have been able to have two children before hysterectomy.^12^ In the UK, Womb Transplant UK (WTUK) announced in February 2025 the safe delivery of the first baby born in the UK following a uterus transplant from a living donor, and the first birth after a deceased donation in December 2025.^13^

Within the UK, UTx is a highly regulated area of medicine despite not being in the contemplation of legislators prior to enactment of the relevant legislation. The Human Fertilisation and Embryology Act 1990, amended by the Human Fertilisation and Embryology Act 2008, was enacted to ‘make provision in connection with human embryos and any subsequent development of such embryos; to prohibit certain practices in connection with embryos and gametes; to establish a Human Fertilisation and Embryology Authority…’ (hereafter the HFEA). The HFEA regulates and licences clinics that provide assisted reproduction. Therefore, in the process of UTx, the HFEA regulates all aspects involving human embryos and gametes, from gamete retrieval, embryo creation, storage, and embryo transfer. The use of assisted reproduction is required for pregnancy attempts, as the recipients’ fallopian tubes are not connected to the donated uterus, thus preventing natural conception. As assisted reproduction is required to attempt to achieve the goal of gestation, the HFEA is involved, as it is a criminal offence to procure, test, process, or distribute gametes without an appropriate licence from the HFEA.^14^ As the number of uterus transplants in the UK is small, established assisted reproduction clinics will be used.

The Human Tissue Act 2004 provides a ‘legislative framework for issues relating to whole body donation and the taking, storage and use of human organs and tissue’.^15^ The statute established the Human Tissue Authority (hereafter the HTA) to oversee organ donation and transplantation and to implement the legislative provisions within, including the consent requirements for organ donation and transplantation. Some 20 years since its enactment, the default position in England and Wales for organ donation is deemed consent; unless a person has recorded a decision not to donate or is in an excluded group, a person is considered to have consented to organ donation.^16^ First implemented in Wales in 2015, followed by England in 2020, deemed consent is applied to specific organs; however, the uterus is expressly excluded from the system of deemed consent.^17^ Notwithstanding this, the statutes do allow for the donation and transplantation of organs and tissues that are not automatically included under the deemed consent system, so the uterus can be legally donated by either a living or a deceased donor. If the donor is alive, express consent is unproblematic (provided that the donor has capacity to consent), and consent to deceased donation of the uterus can either be expressly given by the donor prior to death, by an appointed person, or by someone in a qualifying relationship with the deceased.^18^ Importantly, the Human Tissue Act 2004 does not require organ recipients to be a particular sex or gender in order to receive an organ, and donors are not allowed to require a potential recipient to be a particular sex before donation.

While acknowledging that the process of uterus donation/transplantation and assisted reproduction is well regulated, with statute and regulatory bodies overseeing different parts of the process, there are still pertinent legal and ethical issues that are worthy of detailed examination, as this novel reproductive technology raises issues that were not in the contemplation of Parliament at the time of debating the relevant legislation. It is in light of the recent UK Supreme Court judgment in *For Women Scotland* that, in the next section, I analyse whether access to UTx can legally be expanded to transgender women and if so, whether the necessary fertility treatment can legally be provided to a transgender woman to achieve the reproductive purpose of UTx.

## III. UTx: for (cis) women only?

### A. Selection criteria

The selection criteria for UTx trials worldwide have previously been reviewed by this author with assistance from a research assistant. For interested readers, Supplementary Appendix Table S1 shows the results of this research, detailing the countries that have performed, are currently performing, or are planning to perform uterus transplants. Eligible recipients are highlighted along with the selection criteria focussing upon gender, age limit, and whether the potential recipient needs a partner. As Diaz and others have noted, ‘…although general guidelines are in place, no comprehensive international or national recipient eligibility criteria for UTx are available’.^19^ Despite this, centres conducting UTx (whether as part of a trial or as a treatment for AUFI) have comparable criteria as demonstrated in Supplementary Table S1. The one criterion that all centres have in common is that all ‘…reported cisgender women of reproductive age with a diagnosis of absolute uterine factor infertility as inclusion criteria…’.^20^

The ability to access UTx is therefore currently limited to cisgender women with AUFI who have ovaries, thereby allowing these women to utilize their eggs in the IVF process to have genetically related children.^21^ As can be seen in Supplementary Table S1, this criterion is and has been applied in all uterus transplants performed so far, as well as in the currently recruiting trials and treatment centres in the USA. This therefore prevents access to UTx by cisgender women with AUFI with no ovaries, as well as transgender women. There has been discussion around transgender women accessing uterus transplants, with more limited discussion about this procedure subsequently being extended to cisgender men; however, no surgeon has yet performed a uterus transplant on anyone other than a cisgender woman with AUFI with ovaries. There is discussion around expanding the criterion to be more inclusive (see below), and it is in light of this that I now analyse the legal position of transgender women accessing UTx in the UK.

### B. Transgender women

#### Desire

It must be recognized that without amending the selection criteria to access UTx, anyone who is not a cisgender woman with AUFI and ovaries will be unable to access this reproductive revolution. This includes cisgender women with AUFI who do not have ovaries or who may have an insufficient ovarian supply. I have previously argued that in countries where the use of donated gametes is permitted in assisted reproduction, the selection criteria could and should be expanded to include this group of cisgender women.^22^ It is also recognized that the removal of this criterion could open up UTx to people other than cisgender women.

There has been limited discussion around performing uterus transplants in transgender women for solely gender realignment purposes,^23^ and the medical, social, ethical, and personal value of performing UTx for gender realignment reasons is an important discussion to have; nevertheless, it must be recognized that the possibility of a uterus transplant being performed for this purpose is considered to be almost negligible. There are a limited number of uteri available for donation, both living and deceased, and this scarce resource would be directed to those who wish to fulfil the reproductive role of the uterus.^24^ It is difficult to justify performing a uterus transplant with no intention to attempt to fulfil its reproductive role. The risks and benefits of UTx are difficult to balance; UTx is highly invasive, time-consuming, and expensive to perform and is also temporary. A uterus transplant is a medically complex procedure with many possible complications and health risks for the recipients—the surgical risks as well as short- and long-term impacts of taking immunosuppressive medication are likely to outweigh the benefit of having a uterus solely for gender realignment reasons. As Richards et al. have noted, ‘For TG [transgender) women who wish to preserve their transplanted uterus indefinitely with the immunosuppression for the purpose of menstruation and enhanced gender alignment, the risk-to-benefit ratio is unclear and potentially worrisome.’^25^ That is not to say that the potential recipients would not find value in those experiences, but that the risks involved would be unlikely to justify the short-term benefit.

Finally, the funding of UTx remains unresolved; currently within the UK, WTUK is funding its trial through fundraising as a charity, and the medics involved are donating their time for free. This is not sustainable long-term if, or more likely when, UTx moves towards treatment for AUFI in the UK. Whether there should be public funding of this reproductive technology is a debate that has not yet been resolved,^26^ although if it were to occur, it can be foreseen that the funding would only be available to those who were pursuing UTx for reproductive reasons. It may be that UTx for gender realignment is offered privately, with recipients paying for treatment. This would avoid any legal issues (as discussed in the next section) as fertility treatment would not be provided to someone who was accessing UTx solely for gender realignment purposes, although there would still be a shortage of suitable donors. Considering the likely barriers to access UTx for gender realignment, and the increasing evidence that there is a desire for UTx for reproductive purposes amongst transgender women,^27^ the focus of this article is upon performing UTx for reproductive purposes.

#### Medical barriers

Utilization of uteri by transgender women, and subsequently cisgender men, has been raised by the media and is slowly gaining traction academically.^28^ The possibility of performing a uterus transplant on transgender women and the perspectives of transgender men being potential donors for UTx have also been explored.^29^

It is important to recognize that there is discussion around whether it is medically feasible to perform a uterus transplant in a biologically male body. Jones et al. have noted that

Uterus transplant for transgender women is more complex than for women categorized as female at birth and is currently a prospect with unproven feasibility requiring further research. A number of anatomic, hormonal, fertility, and obstetric issues contribute to the associated increased complexity, but no overwhelming clinical argument can be found that contradicts the feasibility of uterus transplant in this model.^30^

Sampson et al. have provided further detail on these complexities, noting the potential for harm to both the transgender recipient and any foetus due to the greater risks involved with taking hormone medications, the need for transgender recipients to have a neovagina created before a uterus transplant could take place (as clinicians can only access the uterus via the vagina for the purposes of embryo implantation, and to biopsy the cervix to monitor for rejection), and the possible need to undertake surgery to reshape the pelvis to ‘accommodate an additional organ and future fetal growth’.^31^ They note that ‘…the procedure would likely impose substantially elevated risks to genetically XY women above and beyond the risks of UTx for genetically XX women.’^32^ Therefore, they conclude that given ‘…the potential additional risks to genetically XY women, the overall risks may outweigh the benefits at this time….it is premature to recommend the procedure to genetically XY women.’^33^ While many clinicians have recognized the medical complexity and the risks involved, there are some who may still consider performing it. Indeed, in 2022 a clinician in India stated that they planned to perform a uterus transplant in the body of a transgender woman, although no reports have yet confirmed if this occurred.^34^

As Supplementary Appendix Table S1 demonstrates, it is evident that all clinical teams have selection criteria that exclude transgender women automatically, due to the requirement that potential recipients must be able to produce oocytes for use in IVF and subsequent embryo transfer. Terminology such as ‘biologically female’, ‘genotypical female’, ‘female with AUFI’, and ‘female with AUFI and good ovarian reserve’ automatically excludes anyone who does not have ovaries.^35^ Therefore, this excludes not only transgender women, but also cisgender women who do not have ovaries or have an insufficient ovarian reserve; both groups of potential recipients would need to use donated oocytes for IVF treatment. The reproductive goal of UTx is therefore clear, to provide an opportunity to gestate a biologically related child. However, without clear medical justification for excluding the use of donated gametes by a uterus transplant recipient, this will become legally problematic as UTx trials progress to medical treatment in countries, such as the UK, that allow the use of donated gametes in assisted reproduction.^36^ Nevertheless, the possibility of ‘fourth party’ reproduction ‘raises important questions about the ethical, legal and social implications of UTx in TG [transgender] patients,…’.^37^

#### Law and ethics

Notwithstanding the medical feasibility of implanting a uterus in a body that is not biologically female, it is vital that the legal and ethical issues are explored *before* this procedure is performed in transgender women, not least so that the clinicians and recipients are clear where the legal boundaries of their actions lie. It is recognized that transgender access to medical treatment, and more generally transgender rights, is currently a politically contentious issue, so any possible legislative reform is unlikely in the foreseeable future. Nevertheless, history shows us that medicine innovates and progresses faster than the law, and it would be irresponsible and foolish to not consider these issues before science progresses to the point of possibility.

It is recognized that the current heteronormative approach to the accessibility of uterus transplants will be required in some countries in order to remain legally compliant; performing a uterus transplant on someone other than a cisgender woman may be legally prohibited, and/or access to IVF may be limited to heterosexual couples,^38^ thereby making a uterus transplant’s reproductive role redundant if transplanted into a transgender woman. The recent decision in the UK requires a re-examination of the UK legal approach to UTx in transgender women.^39^

#### Equality Act 2010, For Women Scotland, and the Gender Recognition Act 2004

Within the UK, the Equality Act 2010 (the EA) includes gender reassignment as a protected characteristic; the Act outlaws indirect and direct discrimination due to this characteristic.^40^ In this context, in 2019, Jones and others stated that ‘UK and EU legislation would make it legally impermissible to refuse to perform UTx in transgender women solely because of their gender identity’.^41^ Although still subject to ethical scrutiny and HTA approval, a uterus transplant into transgender women’s bodies appears to be legally permitted, and would in fact be subject to a discrimination claim if refusal was solely due to their gender. In light of the recent decision by the UK Supreme Court in *For Women Scotland*, this view is worth revisiting to determine whether someone other than a cisgender woman could first receive a uterus transplant, and second, legally receive fertility treatment to assist them to gestate.

The UK Supreme Court was tasked with determining the statutory interpretation of the terms ‘man’, ‘woman’, and ‘sex’ in the Equality Act 2010. The appellants, a group called For Women Scotland, argued that the definition of a ‘woman’ under the Equality Act 2010 referred to ‘biological sex’, which thereby excluded a transgender woman with a Gender Recognition Certificate (hereafter GRC) from the Equality Act definition of a woman. The respondents, the Scottish Ministers, argued that the definition of a ‘woman’ under the Equality Act 2010 referred to ‘certificated sex’, thereby including a transgender woman with a GRC. This conformed with the longstanding opinion of the Equality and Human Rights Commission.^42^

The Supreme Court had to consider the relevant provisions of the Equality Act 2010 in light of the Gender Recognition Act 2004. Under section 9(1) of the Gender Recognition Act 2004, it is stated that:

Where a full gender recognition certificate is issued to a person, the person’s gender becomes for all purposes the acquired gender (so that, if the acquired gender is the male gender, the person’s sex becomes that of a man and, if it is the female gender, the person’s sex becomes that of a woman).

Further, section 9(3) of the Gender Recognition Act 2004 states that:

Subsection (1) is subject to provision made by this Act or any other enactment or any subordinate legislation.

The Supreme Court noted that there was no provision within the Equality Act 2010 that expressly addressed the effect (if any) of section 9(1) of the Gender Recognition Act 2004 upon the definition of ‘sex’, ‘woman’ or ‘man’ within the Equality Act 2010.^43^ The Supreme Court undertook an analysis of prior legislation, the Sex Discrimination Act 1975 (SDA 1975) and the Sex Discrimination (Gender Reassignment) Regulations 1999, which the Equality Act 2010 ultimately repealed and replaced. The court held that prior to the Gender Recognition Act 2004 there was ‘…no doubt that references to sex, man and woman in the SDA 1975 were references to biological sex’.^44^ The Supreme Court further held that the introduction of the Gender Recognition Act 2004 did not amend the definition of sex or introduce a differently constructed concept of sex.^45^ Finally, the Court had ‘…no reason to suppose that Parliament intended, by the EA 2010, to introduce a change in substance from the SDA 1975, by introducing a modification to the meaning of sex in accordance with section 9(1) of the GRA 2004’.^46^ This has been criticized by Wigley for failing to engage fully with the decision of the House of Lords in *Chief Constable of West Yorkshire v A (no. 2)* [2004] UKHL 21, where it was held that the SDA 1975 did have a trans-inclusive definition of sex.^47^

This reasoning ultimately led the Supreme Court to agree with the position of the appellants, holding that the terms ‘man’, ‘woman’, and ‘sex’ in the Equality Act 2010 referred to biological sex.^48^ As such, transgender people can make a sex discrimination claim on the basis of their biological sex.^49^ The Supreme Court also emphasized that transgender people have the protected characteristic of gender reassignment under the Equality Act 2010, and could also bring claims for direct and indirect discrimination.^50^

The Supreme Court stated that this was necessary for the Equality Act 2010 to be coherent and workable and that any other intention around the definitions within the Act was within the remit of Parliament to alter. This appears to leave transgender people in legal limbo under the Equality Act 2010, only able to access certain services in accordance with their biological sex despite having a GRC that recognizes their acquired sex as their legal sex. Therefore, a transgender woman with a GRC would have their female status legally recognized and yet would not be able to access any services that are designed for women, as this was defined by the court as biological women.

There is also the potential for the *For Women Scotland* decision to impact legal parenthood. Within the Gender Recognition Act, with respect to parenthood, section 12 states that, ‘The fact that a person’s gender has become the acquired gender under this Act does not affect the status of the person as the father or mother of a child’. Brown and Dunne argue that, due to the gendered language used in the parenthood provisions of the HFE Act 2008, ‘based upon the Supreme Courts reasoning…it is presumed that words should have a single and consistent meaning…resulting in ‘woman’ and ‘man’ in the HFEA 2008 being interpreted as ‘biological woman’ and ‘biological man’. If that is the case, and if the gendered terms in the HFEA 2008 are grounded in biology, then there is no role for s12 of the GRA 2004 in determining the parental status of trans people under the ‘parenthood provisions’ of the 2008 statute’.^51^ This ruling ignores the potential for novel reproductive practices, such as UTx, to challenge and change legal understandings of parenthood. As Romanis and Brown have stated, ‘…trans parenthood has altered the conventional understanding by creating circumstances in which the law recognizes male “mothers” and female “fathers”’.^52^ The judgment was concerned with the terms contained within the Equality Act 2010 only, and how these should be interpreted in light of the Gender Recognition Act 2004; the definition of the terms ‘sex’, ‘man’ and ‘woman’ within other statutes were not considered by the court; however, the Supreme Court’s approach is likely to be followed when interpreting and applying those terms as contained within other statutory instruments. As the court stated,

…section 9(1) applies unless section 9(3) applies. Section 9(3) will obviously apply where the GRA 2004 or subsequent enactment says so expressly. But express disapplication of section 9(1) is not necessary as we have explained. Section 9(3) will also apply where the terms, context and purpose of the relevant enactment show that it does, because of a clear incompatibility or because its provisions are rendered incoherent or unworkable by the application of the rule in section 9(1).^53^

Of particular relevance to UTx is the Supreme Court’s position with respect to pregnancy and maternity within the Equality Act 2010. The Supreme Court stated that:

a strong indicator that the words “sex”, “man” and “woman” in the EA 2010 have their biological meaning (and not a certificated sex meaning) is provided by sections 13(6), 17 and 18 (which relate to sex, pregnancy and maternity discrimination) and the related provisions. The protection afforded by these provisions is predicated on the fact of pregnancy or the fact of having given birth to a child and the taking of leave in consequence. *Since as a matter of biology, only biological women can become pregnant, the protection is necessarily restricted to biological women*.^54^ (emphasis added)

While this may currently be true, because clinical trials have excluded transgender women so far, this may change in the very near future. UTx opens the door to the possibility of gestation by people assigned male at birth. If deemed medically feasible and safe to perform, transgender women (who are biologically male) and cisgender men could access UTx, to undergo pregnancy and give birth to a child. Romanis and Brown note that ‘UTx could be utilised to remove the connection between female biological assigned sex and gestation, which is understood as inherent by the law’.^55^ As noted above, the provision of a uterus transplant to a transgender woman (and subsequently cisgender men) in the UK will require careful legal consideration (alongside further medical, ethical, and social considerations) before it proceeds. However, a uterus transplant into a transgender woman’s body could occur elsewhere in the world, before the UK, thereby further negating the Supreme Court’s point.

#### Human rights

Although there is mention in the judgment of *For Women Scotland* of the European Court of Human Rights judgment in *Goodwin v UK* (2002) 35 EHRR 18 in which the UK was held to be in breach of Article 8, the UK Supreme Court judgment lacks an in-depth discussion of human rights, and the impact of the court’s judgment on transgender people’s human rights.^56^ Articles 8 and 14 of the European Convention on Human Rights (ECHR) are of relevance, as are sections 3(1) and 6(1) of the Human Rights Act 1998. Article 8 states ‘Everyone has the right to respect for his private and family life, his home and his correspondence.’ There is a history of legal recognition of transgender people at the highest level. This includes legal recognition of acquired gender,^57^ and overturning an absolute prohibition on trans women marrying their non-trans male partners.^58^ Article 8 has also been used to determine claims by cisgender claimants to artificial insemination facilities^59^ and genetic parenthood.^60^ Alghrani has concluded that these cases demonstrate ‘…that any outright prohibition on uterus transplantation to *restore* fertility and natural function in cisgender women lacking a functioning uterus so as to permit genetic reproduction would engage Article 8’.^61^ Alghrani further notes that ‘It is not clear why the claim of transgender women to gestate is regarded as ‘weak’ or any weaker than that of cisgender women.’^62^ In contrast, Romanis notes that ‘…the ECtHR has never encompassed means of gestation as falling within the realms of procreative decision-making that must be respected as a matter of private life’.^63^ Despite this lack of legal recognition by the European Court of Human Rights that Article 8 encompasses a right to gestate, if there are conditions that prevent access to UTx that impact only certain groups, then there could be a violation of Article 8 or Article 14 (non-discrimination) rights. The failure of the UK Supreme Court to engage meaningfully with human rights leaves scope for further legal challenges.

Wigley has noted that the court has failed in its duty under section 3(1) Human Rights Act 1998 to interpret the Equality Act 2010 in a way that is compatible with the ECHR and that if it felt unable to do so, it should then have made a declaration of incompatibility. This would have forced Parliament to consider and rectify the issue.^64^ The application of human rights to transgender individuals was raised by Amnesty International in their written submissions to the UK Supreme Court, so it is somewhat surprising that the Court has failed to address the potential human rights issues within its judgment.

As Wigley has noted, ‘The judgment has introduced great uncertainty into the legal regime by appearing to endorse trans-exclusionary practices while leaving the human rights implications of such practices at large.’^65^ The case has also been criticized by a group of UN experts who are part of the Special Procedures of the United Nations Human Rights Council, who have warned that the judgment ‘risks entrenching legal uncertainty and undermining the rights of transgender persons in all aspects of life…’.^66^ It is evident that the lack of consideration of the human rights issues has opened up the Supreme Court judgment to criticism and further legal challenge. Indeed, a legal challenge in the European Court of Human Rights alleging violation of Articles 6, 8, and 14 has been initiated.^67^ However, until such point as the European Court of Human Rights hears arguments and gives judgment on this issue, alongside domestic cases which will also seek clarification on the ruling in *For Women Scotland* and the human rights implications, we are left to apply the Supreme Court judgment.

#### Impact on the Human Tissue Act 2004

With regard to receiving a uterus transplant, there is nothing within the Human Tissue Act 2004 that requires an organ transplant recipient to be a specific sex, and so it is argued that transgender women should not be denied a uterus transplant solely due to their sex or gender. They are also still protected under the Equality Act 2010 due to the protected characteristic of gender reassignment. Indeed, Alghrani has argued that the moral right of procreative liberty encompasses the right to gestate and that due to a lack of persuasive evidence to the contrary, the right to gestate as a procreative liberty extends to all individuals, irrespective of sex or gender.^68^ However, providing a uterus transplant to someone other than a cisgender woman now needs to be considered in light of the Supreme Court decision. Although the provision of a uterus to anyone regardless of sex is permitted, the interplay and interaction between organ donation/transplantation and assisted reproduction medicine, along with the relevant statutes, cannot be ignored. UTx for reproductive purposes is effectively a two-stage process: first, organ donation/transplantation as regulated by the Human Tissue Act 2004, and second, use of IVF to facilitate gestation as regulated by the Human Fertilisation and Embryology Act 1990 (as amended), which requires that the provision of ‘treatment services’ is for ‘the purpose of assisting women to carry children’.^69^

## IV. UTx: legal fertility treatment?

As determined above, a transgender person cannot be denied access to a uterus transplant; however, this is not the end of the journey. UTx involves the use of IVF, as the fallopian tubes and ovaries of the biologically female recipient are not connected, thereby negating natural conception. The use of IVF for a transgender woman is further necessitated by the lack of fallopian tubes to connect, although a transgender woman could be a biological parent if they have cryopreserved sperm prior to gender reassignment surgery. In this section, I consider and analyse the legality of providing fertility treatment to a transgender woman to assist them to gestate.

### *McConnell*, the Provision of Treatment Services, and *For Women Scotland*

*R (on the application of TT) v The Registrar General for England and Wales* [2019] EWHC 2384 (Fam) (hereafter *McConnell*) was heard before the President of the Family Division, Sir Andrew McFarlane, who raised the legality of providing ‘treatment services’ to someone other than a cisgender woman. The facts of the case were that McConnell, who had been registered as female at birth, transitioned to live in the male gender and successfully applied for a gender recognition certificate (GRC) under the Gender Recognition Act 2004. McConnell had retained his female reproductive organs, and shortly after the GRC was issued (which granted him male status legally), McConnell underwent intrauterine insemination (IUI) at a licensed clinic; he successfully gestated and gave birth. The issue before the court was whether McConnell should be registered on his child’s birth certificate as ‘mother’ or as the child’s ‘father’, or otherwise ‘parent’ or ‘gestational parent’.^70^ The court held that McConnell was the child’s legal mother, not father, and would appear as such on the child’s birth certificate on the basis that McConnell had gestated and given birth, and it was this that granted legal motherhood, both at common law and within the statute.

While Sir Andrew McFarlane raised the question, the legality of providing ‘treatment services’ to someone other than a cisgender woman was not addressed explicitly by the court, this was due to the fact that ‘…the legality of the treatment [was] not an issue in the present claim. It is, however, a point that the HFEA, the Government and those interested in these matters in Parliament may wish to consider further in the interests of legislative clarity’.^71^ Further, he stated that ‘…there [was], in my view, some doubt that the treatment was lawfully provided under the HFEA regime’.^72^ Equally, the Court of Appeal was also unwilling to consider the issue, stating that ‘…we do not consider it necessary or appropriate to comment on the question whether treatment was lawfully provided under the HFEA 1990 and HFEA 2008…’.^73^

Neither the HFEA nor the Government have undertaken consultative action on this point although the HFEA did subsequently amend its Code of Practice to specifically address the legal parenthood of transgender patients:

The Gender Recognition Act 2004 sets out the circumstances in which a gender recognition certificate (GRC) will be issued and provides trans people with a formal mechanism by which they can be legally recognised in their acquired gender. The centre should be aware that obtaining a GRC (or an interim GRC) does not affect the status of the person as the legal mother, father or second parent of a child. What is relevant in determining legal parenthood is the birth gender of the trans patient.^74^

The HFEA is therefore principally concerned with the legal parenthood of transgender people seeking infertility treatment at licensed clinics, it has not engaged with or addressed the question whether ‘treatment services’ can be provided to someone who is not biologically female, or who has legally acquired male status, and subsequently, whether embryos may be lawfully transferred to them.^75^ Within section 2 of the HFE Act 1990 (as amended), the provision of ‘“treatment services” means medical, surgical or obstetric services provided to the public or a section of the public for the purpose of assisting women to carry children’.^76^ The definition or interpretation of the term ‘woman’ is fundamental in applying the HFE Act to the provision of fertility services to transgender women who have had a uterus transplant; it is also argued that this is also vital for the provision of fertility services to transgender men who have retained their female reproductive organs. Prior to the decision in *For Women Scotland*, I have previously argued that the provision of treatment services to transgender men with GRC’s was unlawful as they were no longer legally recognized as female; Dunne and Brown also supported this position.^77^

Without Parliamentary action, it will be left to the courts to interpret and apply the legislation to determine if treatment services and the transfer of human embryos to assist someone to carry a child can legally be provided to someone other than a cisgender woman. Sir Andrew MacFarlane also noted that there was ambiguity about ‘…the interrelation between the Equality Act and the HFEA and the extent to which treatment of a male person as a ‘woman’ is, or is not, within the statutory scheme entrusted to the Human Fertilisation and Embryology Authority by Parliament’.^78^ In light of the decision in *For Women Scotland*, it is pertinent to consider how these legislative sections may now be interpreted.

Interestingly, in *For Women Scotland*, the justices also considered the *McConnell* case, in the context that limiting the application of section 9(3) to legislation enacted after the Gender Recognition Act 2004 could produce adverse results for the trans community.^79^ As Dunne and Brown have noted, the consideration of *McConnell* is ‘undoubtedly obiter dicta,…[but [while these comments are obiter, they have undoubtedly been included in the judgment for a deliberate purpose’.^80^ However, the Supreme Court refused to express any view on the issues raised in *McConnell* about the legality of providing treatment services to a man. The court only noted that the effect of section 9(1) GRA 2004 on a statute referring to men and women, whether enacted before or after the GRA 2004, ‘…must be carefully considered in light of the wording, context and policy of the statute in question’.^81^ The court further emphasized that ‘section 9(1) applies unless section 9(3) applies’ and the applicable test was that

express disapplication of section 9(1) [was] not necessary…Section 9(3) will also apply where the terms, context and purpose of the relevant enactment show that it does, because of a clear incompatibility or because provisions are rendered incoherent or unworkable by the application of the rule in section 9(1).^82^

This leaves the courts and the regulator, the HFEA, in a position whereby the impact and the definitions of ‘man’ and ‘woman’ as contained within the Equality Act 2010 upon the Human Fertilisation and Embryology Acts are undecided, alongside the Supreme Court ratio requiring the impact of section 9(3) GRA 2004 to be assessed in the terms, context and purpose of the statute. We can consider the approaches that the court may take when this issue undoubtedly comes before the judiciary for legal determination.

### The applicable test

First, looking at the *terms* of the statute, the term ‘woman’ is used throughout the statute, yet there is no general definition of the term. Only within section 3ZA of the HFE Act 2008 are ‘woman’ and ‘man’ defined for that particular section. Examples within the statute (as originally enacted) include:

- section 2(3) ‘For the purposes of this Act, a woman is not to be treated as carrying a child until the embryo has become implanted’.
- section 3(2) ‘No person shall place in a woman – (a) a live embryo other than a human embryo…’
- section 4(1)(b) ‘No person shall in the course of providing treatment services for any woman…’
- section 13(5) ‘A woman shall not be provided with treatment services unless account has been taken of the welfare of any child who may be born as a result of the treatment…’
- section 27 (1) ‘The woman who is carrying or has carried a child as a result of the placing in her of an embryo or of sperm and eggs, and no other woman, is to be treated as the mother of the child’.
- section 27 (3) ‘Subsection (1) above applies whether the woman was in the United Kingdom or elsewhere at the time of the placing in her of the embryo or the sperm and eggs.’
- schedule 2 paragraph 1(1)(e) ‘A licence under this paragraph may authorise any of the following in the course of providing treatment services—placing any embryo in a woman…’

The courts could choose to apply the literal approach and interpret ‘woman’ as understood in the late 1980’s when the HFE Act was drafted; this would correspond with the now determined Equality Act definition of ‘woman’ as referring to a biological woman. As only biological women were known to be able to gestate in the 1980’s, it is difficult to come to another conclusion considering that at the time it was unheard of for transgender men to gestate. The HFE Act 1990 was subsequently amended following the enactment of the HFE Act 2008; however, no changes to the above sections were made that impacted the understanding of woman as enacted in 1990. What has been introduced (and as discussed below) is a definition for embryos and gametes, and from whom these may be extracted for use in fertility treatment. In addition, Parliament did not (to the best of my knowledge) debate the amendments with reference to the Gender Recognition Act 2004, and its potential impact.

Second, if we look at the *context* in which the HFE Act 1990 was drafted and subsequently enacted, there were concerns about the ‘wild west’ of IVF, the lack of regulation of new assisted reproductive technologies, and the impact upon women and resulting children. Medical and societal understandings of the time were that only biological women could gestate and give birth to children. This is evidenced in the language of the statute in that it uses the term ‘woman’ consistently and in all contexts, relating to embryos, the placing of an embryo, and providing treatment services to assist women to carry children. In the 1980s, it was understood that only biological women with a uterus could gestate; there are no references to men or someone other than a biological woman gestating. If Parliament had intended to include the provision of fertility services to enable anyone to gestate, we would expect to see either gender neutral language, such as ‘assisting people to carry children’, or for definitions to be provided for those eligible.

If the courts choose to read the legislation within the societal and medical context in which it was drafted, whereby only biological women can gestate, this could then be applied to transgender men to allow them to access fertility services to assist them to carry a child, by viewing them as ‘female’ as this is their biological sex and would be in accordance with the Supreme Court decision. This would, however, go against the desires of the transgender community; for some, it would also go against their legally recognized gender and was notably a point of contention in *McConnell*; counsel for the government argued that ‘trans-men’ are treated as ‘women’ in order to come within the provisions of the HFEA legislation’, whereas counsel for McConnell and his child, YY, refuted that position and argued that ‘under the Equality Act 2010, clinics must offer services to both women and men’.^83^ From a legal perspective, this point was disappointingly not developed by counsel or determined by the court, as the legality of the treatment provided was not part of McConnell’s legal claim. It is also noted that transgender men are registered as the child’s legal mother, due to the fact of gestation. As Brown has noted, ‘…the legislative approach to the attribution of legal parenthood has consistently failed to properly account for those families whose lived reality is situated outside the confines of the traditional, heterosexual, nuclear family.’^84^ This strict literal interpretation would also be applied to transgender women whereby they would be considered to be ‘male’ for the purposes of fertility treatment in accordance with their biological sex, this would prevent transgender women from falling into this definition of ‘woman’ as they had been assigned male at birth. It would then prevent transgender women from accessing treatment services that are designed to assist women to carry children. Therefore, if a transgender woman was in receipt of a uterus transplant, they could not legally access services designed to assist (biological) women to carry children, as they are not biologically female. As Dunne and Brown have noted, ‘it appears unlikely that a future court would consider that this legislative context would indicate an intention for these terms to have a variable definition.’^85^ The preferable approach would be to recognize transgender people in accordance with their certificated sex, but this approach was not favoured by the UK Supreme Court.

Third, the court must consider the *purpose* of the legislation at the time of enactment (also known as the purposive approach to statutory interpretation). The purpose of the Human Fertilisation and Embryology Act 1990 is:

An Act to make provision in connection with human embryos and any subsequent development of such embryos; to prohibit certain practices in connection with embryos and gametes; to establish a Human Fertilisation and Embryology Authority; to make provision about the persons who in certain circumstances are to be treated in law as the parents of a child; and to amend the Surrogacy Arrangements Act 1985.^86^

As the purpose of the Act was to regulate the use of human embryos, the court could interpret this to apply to anyone who sought access to fertility treatment so that the definition of ‘woman’ within the Human Fertilisation and Embryology Acts could include transgender women alongside cisgender women, as well as transgender men (as they were born with female anatomy). Alternatively, the court may decide to require transgender women to have a GRC that would confirm their female (legal) status. This would, however, then logically exclude transgender men with a GRC from accessing fertility services to enable them to gestate. This would be contrary to the approach of the Supreme Court, which decided, in the context of the Equality Act 2010, that differentiating between transgender people with and without a GRC (what the court referred to as ‘certificated sex’) was complicated and would make the legislative scheme inconsistent and unworkable. It must be noted that the two cases differ in their approach, not least because they were dealing with two different legal issues: *McConnell* was challenging the definitions of ‘mother’ and ‘father’ within the common law alongside section 12 of the Gender Recognition Act 2004, while *For Women Scotland* was concerned with the impact of section 9(3) of the Gender Recognition Act 2004 on the Equality Act 2010.

Considering the terms and context of the legislation, it appears that transgender women could not access fertility services in order to assist them to carry a child, as this would be contrary to the literal interpretation. However, when considering the purpose of the legislation, there is a lack of clarity and the approach to be taken by the courts is uncertain. Proportionality must also be taken into consideration. The UK Supreme Court considered justifications such as women’s safety as legitimate and proportionate grounds for the provision of single sex services.^87^ If transgender women are able to receive a donated uterus, there would need to be a proportionate and legitimate aim to exclude them from accessing assisted reproduction.^88^ While safety justifications would not apply here, any interference would need to be proportional, and it is predicted will be tested before the courts.

Further uncertainty arises when looking at the HFEA approach to legal parenthood. The HFEA approach to legal parenthood, whereby the birth gender of a transgender patient is determinative of legal parenthood, corresponds with the decision of the Supreme Court in *For Women Scotland*, although it fails to engage with the legality of providing treatment services (ie the process of transferring human embryos or IUI) to someone other than a cisgender woman to assist them to carry a child. Further, the legislation only allows the use of permitted embryos or permitted eggs or sperm to be placed in a woman. Contained within section 3(2) and 3ZA HFE Act 1990 (as amended), it is stated that:

‘No person shall place in a woman—

(a) an embryo other than a permitted embryo (as defined by section 3ZA), or

(b) any gametes other than permitted eggs or permitted sperm (as so defined)’ (section 3(2) HFE Act)

(1) This section has effect for the interpretation of section 3(2).

(2) A permitted egg is one—

(a)which has been produced by or extracted from the ovaries of a woman, …

(3) Permitted sperm are sperm—

(a)which have been produced by or extracted from the testes of a man, …

(4) An embryo is a permitted embryo if—

(a) it has been created by the fertilisation of a permitted egg by permitted sperm,

…

(6) In this section—

(a) “woman” and “man” include respectively a girl and a boy (from birth), …

(section 3ZA HFE Act)

It is therefore explicitly stated that permitted eggs must have been ‘produced by or extracted from the ovaries of a woman’, and permitted sperm must have been ‘produced by or extracted from the testes of a man’. It is the additional requirement that ‘“woman” and “man” include respectively a girl and a boy (from birth)’ which needs to be carefully interpreted and applied. A purposive interpretation may be difficult to argue due to the inclusion of ‘from birth’ which indicates a continuing status. A purposive interpretation would also go against the approach of the Supreme Court in which continuing biological sex was determinative. Permitted eggs and permitted sperm would therefore appear to be only those that are extracted from women and men who are the gender that they were assigned at birth and have maintained that gender at the point of production/extraction.^89^ Only then can they use them in any fertility treatment that they are accessing. This would appear to prevent the use of transgender men’s oocytes and transgender women’s sperm in the creation of embryos unless they were extracted prior to their gender transition. Gamete extraction must have occurred prior to gender transition, but they could not be used in subsequent fertility treatment unless that treatment is being provided to a biological woman to assist her to gestate. I have previously argued that this ‘prohibits the transfer of human embryos to anyone other than a woman who has been a woman from birth.’^90^ The continuing status from birth to assisted reproductive treatment and beyond corresponds with the biological view adopted by the Supreme Court. Romanis agrees that ‘if interpreted literally [s3ZA] has the effect of rendering it unlawful to use the eggs of a legally male person (or the sperm of a legally female person)’.^91^

The failure of the HFEA to engage with and to address this important issue was a fundamental flaw in the amendments made to the Code of Practice, and it is predicted that this is now, or will very shortly be, reviewed by the HFEA following the Supreme Court judgment. While it may have dealt with legal parenthood for transgender people who access fertility treatment services, it fails to address the fundamental question as to whether they can be legally provided with treatment services and, as such, whether human embryos can be transferred to someone other than a cisgender woman to assist them to carry a child. Further discussion, debate and clarification of these legislative provisions must be made before UTx becomes medically possible in transgender women (and therefore cisgender men) and is performed within the UK’s legal jurisdiction.^92^

## V. Conclusion

UTx represents one of the most significant developments in reproductive medicine, sitting at the intersection of organ transplantation and assisted reproduction. As a temporary, life-producing transplant performed on otherwise healthy individuals, UTx challenges established legal frameworks that were developed long before such possibilities existed. While the medical feasibility of transplanting a uterus into a transgender woman remains untested, the prospect is no longer purely speculative. Consequently, it is necessary to examine whether existing law is capable of accommodating such developments.

Following the recent decision of the UK Supreme Court in *For Women Scotland*, this article has analysed two distinct but interconnected legal questions: whether a transgender woman may lawfully receive a uterus transplant and whether, following transplantation, fertility treatment may lawfully be provided to enable gestation. In relation to the first question, the position is relatively straightforward. The Human Tissue Act 2004 does not require someone to be a particular sex or gender before receiving an organ transplant. Furthermore, transgender individuals remain protected from discrimination by virtue of the protected characteristic of gender reassignment. Consequently, there appears to be no legal prohibition on the transplantation of a uterus into a transgender woman.

The more difficult legal question arises once the reproductive purpose of UTx is contemplated. UTx cannot be separated from the fertility treatment required to achieve gestation, as pregnancy depends upon the use of assisted reproductive technologies regulated by the Human Fertilisation and Embryology Acts; the legality of the transplant is only part of the story. The statutory framework repeatedly refers to the provision of treatment services for the purpose of assisting women to carry children and regulates the transfer of embryos and use of gametes through language that is highly gendered.

The significance of the Supreme Court’s decision in *For Women Scotland Ltd v The Scottish Ministers* is therefore difficult to overstate. Although the judgment was concerned with the interpretation of the Equality Act 2010, its reasoning places renewed emphasis on biological sex as the meaning of the terms ‘woman’, ‘man’, and ‘sex’ unless a contrary interpretation is clearly required by the relevant statutory scheme. Applying that approach to the Human Fertilisation and Embryology Acts creates substantial uncertainty. A literal and contextual interpretation of the legislation suggests that Parliament envisaged treatment services being provided only to biological women. While purposive arguments may support a broader interpretation, particularly given the evolving nature of reproductive technologies, they sit uneasily alongside the Supreme Court’s reasoning and the statutory language itself. Until the courts clarify the statutory interpretation of ‘providing treatment services to assist women to carry children’ as contained within the Human Fertilisation and Embryology Acts, it may be presumed that clinics providing fertility services will follow the approach that has been set by the Supreme Court, or risk being found to be providing services illegally. It is anticipated that a legal case challenging this position will soon be brought specifically looking at the application of the decision to the Human Fertilisation and Embryology Act 1990 (as amended); the outcome will determine once and for all whether or not transgender women can access UTx for reproductive reasons.

Accordingly, this article argues that the current law produces a paradox. A transgender woman may be able to receive a uterus transplant yet may subsequently be unable to access the fertility treatment necessary to fulfil the reproductive purpose of that transplant. This disconnect reveals the limitations of a regulatory framework designed decades before UTx, transgender gestation, and other emerging reproductive possibilities entered legal and clinical discourse. The resulting uncertainty is unsatisfactory for clinicians, regulators, and prospective patients alike.

Ultimately, the issues examined here extend beyond UTx. They expose broader tensions between rapidly advancing reproductive technologies and legislative schemes rooted in assumptions about sex, reproduction, and parenthood that are increasingly challenged by medical innovation. Whether one supports or opposes the extension of UTx to transgender women, questions concerning access to fertility treatment, the interpretation of sex-specific statutory language, and the legal recognition of novel forms of gestation require clear and principled answers. The current framework provides none. Legislative clarification, informed by medical evidence, human rights considerations, and contemporary understandings of sex, gender, and reproduction, is therefore essential. Until such reform occurs, the legality of reproductive UTx for transgender women in the UK will remain uncertain and contested, with the law struggling to keep pace with the possibilities created by modern medicine.

## Supplementary Material

fwag035_Supplementary_Data

## Footnotes

1 Department of Health and Social Security, *Report of the Committee of Inquiry into Human Fertilisation and Embryology Cmnd 9314* (1984); Department of Health, *The Report of the Public Inquiry into children’s heart surgery at the Bristol Royal Infirmary 1984-1995: Learning from Bristol* (2001); M Redfern, *The Royal Liverpool Children’s Inquiry: Report* (HMSO 2001). The Warnock, Kennedy and Redfern Reports, respectively.

2 For a discussion of how far the legislation following the Warnock Report can be applied to recent reproductive developments, see N Hammond-Browning, ‘Ethics, Embryos, and Evidence: A Look Back at Warnock’ (2015) 23 Medical Law Review 588; Victoria Adkins, ‘The Warnock Report and Partial Ectogestation: Retracing the Past to Step into the Future’ (2023) 31 Medical Law Review 424.

3 Thanks to an anonymous reviewer for raising this point.

4
*R (on the application of TT) v The Registrar General for England and Wales* [2019] EWHC 2384 (Fam); *R (on the application of McConnell and YY) v Registrar General for England and Wales* [2020] EWCA Civ 559.

5 M Brännström and others, ‘Livebirth after Uterus Transplantation’ (2015) 385 Lancet 607.

6 For a thorough analysis of the many issues raised by uterus transplantation, refer to N Hammond-Browning and NJ Williams (eds), *International Legal and Ethical Perspectives on Uterus Transplantation* (Edward Elgar 2024).

7 ibid Introduction.

8 Adapted from N Hammond-Browning and NJ Williams, ‘Introduction’ in N Hammond-Browning and NJ Williams (eds), *International Legal and Ethical Perspectives on Uterus Transplantation* (Edward Elgar 2024).

9 COTS: Childlessness Overcome Through Surrogacy <https://www.surrogacy.org.uk/> accessed 13 March 2025.

10 Brännström and others (n 5).

11 D Ejzenberg and others, ‘Livebirth after Uterus Transplantation from a Deceased Donor in a Recipient with Uterine Infertility’ (2018) 392 The Lancet 2697.

12 M Brännström, L26/O-124 Uterus Transplantation Versus Artificial Uterus (2026) 41 Human Reproduction deag083.124. https://doi.org/10.1093/humrep/deag083.124.

13 ‘A little miracle’: First baby born in UK to woman with transplanted womb. Fergus Walsh, BBC News, 7^th^ April 2025; ‘The Gift of Life’, Womb Transplant UK, 24^th^ February 2026 <https://wombtransplantuk.org/press-release-miracle-baby-boy> accessed 11 June 2026.

14 Human Fertilisation and Embryology Act 1990, s 41.

15 Human Tissue Act 2004, Explanatory Notes, para 4.

16 NHS Blood and Transplant <https://www.organdonation.nhs.uk/helping-you-to-decide/organ-donation-laws/>.

17 Human Transplantation (Wales) Act 2013; The Human Transplantation (Excluded Relevant Material) (Wales) Regulations 2015; Organ Donation (Deemed Consent) Act 2019; The Human Tissue (Permitted Material: Exceptions) (England) Regulations SI 2020/521.

18 Consent for Transplantation Activities Involving Excluded Material, the Human Transplantation (Wales) Act 2013, s 7, Table 4; The Human Tissue Act 2004, s 6.

19 AL Diaz and others, ‘Vascularized Composite Allotransplantation of the Uterus: A Systematic Review of Eligibility Criteria’ (2025) 94 Annals of Plastic Surgery 595, 595.

20 ibid.

21 N Hammond-Browning, ‘UK Criteria for Uterus Transplantation: A Review’ (2019) 126 British Journal of Obstetrics and Gynaecology 1320.

22 ibid.

23 For example, refer to: BP Jones and others, ‘Perceptions and Motivations for Uterus Transplant in Transgender Women’ (2021) 4 JAMA Network Open e2034561; DN Wagner, ‘For Women Only? Reconsidering Gender Requirements for Uterine Transplantation Recipients’ (2023) 6 Canadian Journal of Bioethics = Revue Canadienne de Bioéthique 53.

24 A discussion of the allocation or prioritisation of uteri is beyond the scope of this article; however, interested readers can refer to the work in this area including R Tonkens, ‘Gatekeeping Uterus Transplants: A Proposal for Eligibility Criteria and the Fair Allocation of Wombs’ in N Hammond-Browning and NJ Williams (eds), International Legal and Ethical Perspectives on Uterus Transplantation (Edward Elgar Publishing Limited 2024); R Tonkens, ‘Misusing Uteruses? Childrearing Capacity and Access to Transplantable Wombs’ (2020) 34 Bioethics 105.

25 EG Richards and others, ‘A “First” on the Horizon: The Expansion of Uterus Transplantation to Transgender Women’ (2023) 119 Fertility and Sterility 390, 391.

26 For example, S Wilkinson and NJ Williams, ‘Should Uterus Transplants Be Publicly Funded?’ (2016) 42 Journal of Medical Ethics 559; A Alghrani, ‘Should Uterus Transplants Be Publicly Funded!’’ (2016) 42 Journal of Medical Ethics 566.

27 Jones and others (n 23).

28 A Alghrani, ‘Uterus Transplantation in and Beyond Cisgender Women: Revisiting Procreative Liberty in Light of Emerging Reproductive Technologies’ (2018) 5 Journal of Law and the Biosciences 301; E Bayar and others, ‘Fertility Preservation and Realignment in Transgender Women’ (2023) 26 Human Fertility 463; N Hammond-Browning, ‘ Uterine Transplantation in Transgender Women: Medical, Legal and Ethical Considerations’ (2019) 126 British Journal of Obstetrics and Gynaecology 157.

29 M Carbonnel and others, ‘Transgender Males as Potential Donors for Uterus Transplantation: A Survey’ (2022) 11 Journal of Clinical Medicine 6081.

30 Jones and others (n 23).

31 A Sampson and others, ‘Uterus Transplantation in Women Who Are Genetically XY’ (2019) 45 Journal of Medical Ethics 687.

32 ibid 687.

33 ibid 688.

34 A Shoaib, ‘ Doctor Planning Womb Transplant to Allow a Transgender Woman to Carry Children’ *Business Insider* <https://www.businessinsider.com/india-doctor-plans-womb-transplant-trans-woman-to-carry-children-2022-5> accessed 28th July 2025.

35 Also noted by Diaz and others (n 19) 596.

36 Hammond-Browning (n 21).

37 Richards and others (n 25) 391.

38 eg Italy and the Czech Republic restrict access to IVF to heterosexual couples.

39 For the legal position prior to the decision in *For Women Scotland*, refer to my detailed analysis in N Hammond-Browning, ‘Regulating Uterus Transplantation: The United Kingdom’ in N Hammond-Browning and NJ Williams (eds), *International Legal and Ethical Perspectives on Uterus Transplantation* (Edward Elgar 2024).

40 Equality Act 2010, s 4.

41 BP Jones and others, ‘Uterine Transplantation in Transgender Women’ (2019) 126 British Journal of Obstetrics and Gynaecology 152.

42
*For Women Scotland Ltd v The Scottish Ministers* [2025] UKSC 16, para 19 and 33.

43 ibid para 158.

44 ibid para 162.

45 ibid para 163.

46 ibid para 164.

47 C Wigley, *For Women Scotland: a legal critique* (Translegalproject.org 2025). With thanks to Crash Wigley for raising this point on a draft of this article.

48
*For Women Scotland Ltd v The Scottish Ministers* (n 42) para 264.

49 ibid para 264.

50 ibid para 249–264.

51 A Brown and P Dunne, ‘The Family Law Implications and Consequences of *For Women Scotland*’ (2025) 10 Family Law 1191, 1197.

52 EC Romanis and A Brown, ‘Legal Parenthood, Novel Reproductive Practices, and the Disruption of Reproductive Biosex’ (2025) 88 The Modern Law Review 155, 165–166.

53
*For Women Scotland Ltd v The Scottish Ministers* (n 42) para 156.

54 ibid (n 42) para 177.

55 Romanis and Brown (n 52) 177.

56
*For Women Scotland Ltd v The Scottish Ministers* (n 42) para 63–65.

57
*Goodwin v. the United Kingdom* 35 EHRR 18.

58
*Bellinger v Bellinger* [2003] UKHL 21.

59
*Dickson v UK* [2007] 3 F.C.R. 877.

60
*Hadley v Amicus Healthcare Ltd & Others: Evans v Midland Fertility Services & Others* [2003] EWHC 2161 (Fam).

61 Alghrani (n 28) 10.

62 ibid 20.

63 EC Romanis, *Biotechnology, Gestation, and the Law* (OUP 2025) 87.

64 Wigley (n 47).

65 ibid.

66 UN Human Rights Council Special Procedures, ‘UN Experts Warn of Legal Uncertainty and Rights Implications Following UK Supreme Court Ruling’ (2025).

67 Monckton Chambers, ‘Legal challenge submitted to the European Court of Human Rights in light of recent Supreme Court judgment’ (20 August 2025) <https://www.monckton.com/legal-challenge-submitted-to-the-european-court-of-human-rights-in-light-of-recent-supreme-court-judgment/> accessed 14 July 2026.

68 Alghrani (n 28).

69 Human Fertilisation and Embryology Act 1990, s 2.

70
*R (on the application of TT) v The Registrar General for England and Wales* (n 4).

71 ibid para 22.

72 ibid para 127.

73
*R (on the application of McConnell and YY) v Registrar General for England and Wales* (n 4) para 26.

74 HFEA, *9th Code of Practice* (2019) updated October 2023, para 6.50.

75 For an analysis of the implications of *For Women Scotland* on the parental status of transgender parents, refer to Brown and Dunne (n 51).

76 Human Fertilisation and Embryology Act 1990, s 2.

77 Hammond-Browning (n 39); P Dunne and A Brown, ‘Trans Parenthood in the UK: The "Unanswered Questions" of the McConnell Litigation’ (2024) 83 The Cambridge Law Journal 490.

78
*R (on the application of TT) v The Registrar General for England and Wales* (n 4) para 159.

79
*For Women Scotland Ltd v The Scottish Ministers* (n 42) para 105.

80 Brown and Dunne (n 51) 1195.

81
*For Women Scotland Ltd v The Scottish Ministers* (n 42) para 108.

82 ibid para 156.

83
*R (on the application of TT) v The Registrar General for England and Wales* (n 4) para 22.

84 A Brown, ‘Trans Parenthood and the Meaning of ‘Mother’, ‘Father’ and ‘Parent’ - R (McConnell and YY) v Registrar General for England and Wales [2020] EWCA Civ 559’ (2021) 29 Medical Law Review 157, 169.

85 Brown and Dunne (n 51) 1195.

86 Human Fertilisation and Embryology Act 1990, Introductory text.

87
*For Women Scotland Ltd v The Scottish Ministers* (n 42) para 214.

88 Thanks to one of the anonymous reviewers for raising this point.

89 Hammond-Browning (n 21).

90 ibid 1322.

91 EC Romanis, ‘Mens Pregnancies and Nonbinary Pregnancies in The Law: Ontic Injustice and/or Invisibilization’ (2026) 51 Alternatives: Global, Local, Political 69, 77.

92 Hammond-Browning (n 39).

