# Supplementary material for: The legal paradox of uterus transplantation in transgender women: reproductive innovation and regulatory limits after For Women Scotland
Source: Med Law Rev. 2026 Oct 5;34(4):fwag035. doi: 10.1093/medlaw/fwag035 (PMC13637585; doi:10.1093/medlaw/fwag035)
Supplement: Supplementary file 1 [file fwag035_supplementary_data.docx]

# Appendix

Hannah Furness (medical student, Cardiff University) was employed in the summer of 2022 to conduct this research. Papers and clinical trials for review were found using Pub Med and clinicaltrials.gov, information was also found on clinical teams websites using a simple Google search. The key words used were: ‘uterus transplant’, ‘uterine transplant’, ‘womb transplant’, infertility’ and ‘MRKH’. The word ‘and’ was also used in the advanced search option to combine the search terms for more specific results. These searches were repeated and updated in 2025 by the author.

**Table 1:** Demonstrating the gender, age and relationship requirements of potential uterus transplant recipients

| **Country (date of 1^st^ transplant)** | **Who can access UTx?** | **Age limits** | **Need for a partner?** |
| --- | --- | --- | --- |
| Saudi Arabia (2000)^[1]^ | Selection criteria unknown – 1 UTx performed on a 26yr old female | Unknown | Unknown |
| Turkey (2011)^[2]^ | XX female | Unknown | Unknown |
| Sweden (2013)^[3]^ | Female with AUFI with normal ovarian reserve | <38 years old | In a stable relationship or married |
| China (2015)^[4]^ | Unknown but 1^st^ recipient was a married, female aged 23 | Unknown | Unknown |
| Brazil (2016)^[5]^ | Female | Aged between 21 – 38 yrs | Stable relationship of at least 2 years. (Implied partner is male as partners sperm is analysed) |
| USA, Dallas (2016)^[6]^ | Biologically female (karyotype XX) with AUFI and a good ovarian reserve | 20-30 years | Unknown |
| USA, Cleveland (2016)^[7]^ | Female with AUFI | 21-45 years (embryos must have been produced by age 39) | Unknown |
| Czech Republic^[8]^ | Female with AUFI | 18 to 40 years | Must have a male partner |
| Germany^[9]^ | Female with AUFI with ovaries | <38 years | Unknown |
| USA, Pennsylvania (2017)^[10]^ | Offered as a treatment, not a trial. Criteria unknown | Unknown | Unknown |
| Serbia (2017)^[11]^ | Unknown | Unknown | Unknown |
| India (2017)^[12]^ | Female with AUFI with good ovarian reserve | <35 years | Unknown |
| Lebanon (2018)^[13]^ | Female with AUFI with good ovarian reserve | <38 years | In a stable relationship or married |
| Belgium (2018)^[14]^ | Biological female with MRKH | 18-38 years | To be in a stable relationship of at least one year |
| France, Foch (2019)^[15]^ | Female with MRKH | 18-38 years | Be in a stable couple |
| Italy (2020)^[16]^ | Female with AUFI (acquired or congenital) | 18-40years | In a stable relationship (Implied partner is male as partners sperm is analysed) |
| USA, Alabama (2020)^[17]^ | Female with AUFI | Unknown | Unknown |
| Spain (2020)^[18]^ | Female with AUFI | <40 years | Unknown |
| Australia (2023)^[19]^ | Female with AUFI with intact ovaries | 18-42 years (with embryos frozen prior to age 38) | Unknown |
| United Kingdom (2023)^[20]^ | Female with AUFI | 24-38 years (or 40 if eggs frozen before age 38) | Preference to be in a stable and loving relationship |
| USA, Houston, Texas Children’s Hospital (recruiting)^[21]^ | Biologically female (XX) women with AUFI with at least 1 functioning ovary | 20 - 40 years | Unknown |
| USA, Maryland, John Hopkin’s (recruiting)^[22]^ | Genotypic female with AUFI (own ovaries) | 18-38 years at time of egg retrieval | Unknown |
| France, Rennes (recruiting)^[23]^ | Female with AUFI (MRKH or hysterectomy) | 18-40 years | In a heterosexual relationship, in a same sex relationship with another woman, or single women |

1. W. Fageeh and others, 'Transplantation of the human uterus' (2002) 76 Int J Gynaecol Obstet 245
2. O. Ozkan and others, 'Preliminary results of the first human uterus transplantation from a multiorgan donor' (2013) 99 Fertil Steril 470
3. Brännström M. Uterus Transplantation LD - Feasibility Study - Sahlgrenska - BMC (GOT-BMC-UTx) NCT03590405 <https://clinicaltrials.gov/study/NCT03590405>
4. Malasevskaia I, Al-Awadhi AA. A New Approach for Treatment of Woman With Absolute Uterine Factor Infertility: A Traditional Review of Safety and Efficacy Outcomes in the First 65 Recipients of Uterus Transplantation. *Cureus*. 2021;13(1):e12772-e
5. De A Vieria M. Laparoscopic-robotic Hysterectomy for Uterine Transplantation in Live-donor Patient. <https://clinicaltrials.gov/ct2/show/NCT04249791>; 2020.
6. Testa G, McKenna GJ, Bayer J, Wall A, Fernandez H, Martinez E, et al. The Evolution of Transplantation From Saving Lives to Fertility Treatment: DUETS (Dallas UtErus Transplant Study). *Annals of Surgery*. 2020;272(3); Testa G, Koon EC, Johannesson L, McKenna GJ, Anthony T, Klintmalm GB, et al. Living Donor Uterus Transplantation: A Single Center's Observations and Lessons Learned From Early Setbacks to Technical Success. *American Journal of Transplantation*. 2017;17(11):2901-10
7. Quintini C. Uterine Transplantation for the Treatment of Uterine Factor Infertility <https://clinicaltrials.gov/ct2/show/NCT02573415>; 2015
8. Fronek J, Kristek J, Chlupac J, Janousek L, Olausson M. Human Uterus Transplantation from Living and Deceased Donors: The Interim Results of the First 10 Cases of the Czech *Trial. J Clin Med*. 2021 Feb 4;10(4):586
9. F. A. Taran and others, 'Screening and evaluation of potential recipients and donors for living donor uterus transplantation: results from a single-center observational study' (2019) 111 Fertil Steril 186
10. No criteria publicly available
11. I. Malasevskaia and A. A. Al-Awadhi, 'A New Approach for Treatment of Woman With Absolute Uterine Factor Infertility: A Traditional Review of Safety and Efficacy Outcomes in the First 65 Recipients of Uterus Transplantation' (2021) 13 Cureus e12772. The selection criteria for the transplant in Serbia are not publicly available but may be similar to the Swedish team, as that team led the transplant procedure.
12. S. Puntambekar and others, 'Novel Anastomotic Technique for Uterine Transplant Using Utero-ovarian Veins for Venous Drainage and Internal Iliac Arteries for Perfusion in Two Laparoscopically Harvested Uteri' (2019) 26 J Minim Invasive Gynecol 628
13. Uterus Transplantation LD - Feasibility Study - Sahlgrenska - BMC (GOT-BMC-UTx), Sahlgrenska University Hospital with Bellevue Medical Center affiliated to St Jospeh University, Beirut, Lebanon, NCT03590405 <https://clinicaltrials.gov/study/NCT03590405?id=NCT03590405&rank=1>
14. Uterus Transplantation from a Multi-Organ Donor, University Hospital, Ghent, Belgium, NCT03252795 <https://clinicaltrials.gov/study/NCT03252795>
15. Feasibility Study of Uterine Transplantation From Living Donors in Terms of Efficacy and Safety in Patients With Mayer-Rokitansky-Küster-Hauser Syndrome (MRKH), Hospital Foch, France, NCT03689842 <https://clinicaltrials.gov/study/NCT03689842?cond=Mrkh%20Syndrome&term=uterus%20transplant&rank=6>
16. Diminished ovarian reserve and ectopic ovaries in patients with Mayer-Rokitansky-Küster-Hauser syndrome candidates for Uterus Transplantation: our experience
17. UAB Medicine, ‘Uterus Transplant FAQs’ <https://www.uabmedicine.org/wp-content/uploads/sites/3/2022/12/Uterus-Transplant-FAQs.pdf>
18. F. Carmona and others, 'Uterine transplantation. First viable case in Southern Europe' (2021) 156 Med Clin (Barc) 297; Feasibility Study of Uterus Transplantation Procedure From a Live Donor Obtaining the Graft by Laparoscopy, Hospital clinic of Barcelona, Spain, NCT04314869 <https://clinicaltrials.gov/study/NCT04314869>
19. Rebecca Deans and others, 'The first Australian uterus transplantation procedure: A result of a long-term Australian-Swedish research collaboration' (2023) 63 Australian and New Zealand Journal of Obstetrics and Gynaecology 418
20. BP Jones and others, 'Uterine transplantation: past, present and future' (2016) 123 BJOG: An International Journal of Obstetrics & Gynaecology 1434; Jones B. INvestigational Study Into Transplantation of the Uterus (INSITU). <https://clinicaltrials.gov/ct2/show/NCT04244409> 2020; UK WT. Who is Eligible? Womb Transplant UK; 2022 <https://wombtransplantuk.org/about/who-is-eligible>
21. Uterine Transplantation and Pregnancy Induction in Women Affected by Absolute Uterine Factor Infertility, Texas Children’s Hospital and Baylor College of Medicine, NCT05263076 <https://www.clinicaltrials.gov/study/NCT05263076#participation-criteria>; Texas Children’s Hospital, ‘Uterus Transplant Research Program’ <https://www.texaschildrens.org/departments/uterus-transplant-research-program>
22. OPRTUNTI: Offering Potential for Reproduction Through Transplantation of Uterus iN the Treatment of Infertility, John Hopkins University, Baltimore, USA, NCT05646992 <https://clinicaltrials.gov/study/NCT05646992?id=NCT05646992&rank=1>;
23. Transplantation of Uterus for Uterine infertiLIty From Living Donor or Deceased Donor, Rennes University Hospital, France, NCT05726305 <https://clinicaltrials.gov/study/NCT05726305?locStr=France&country=France&cond=Uterus%20Transplant&rank=2>; Uterine Transplant Program, John Hopkins Medicine <https://www.hopkinsmedicine.org/gynecology-obstetrics/specialty-areas/uterine-transplant#eligibility>
